# Supplementary material for: Development of a prediction model to estimate the 5-year risk of cardiovascular events and all-cause mortality in haemodialysis patients: a retrospective study
Source: PeerJ. 2022 Nov 9;10:e14316. doi: 10.7717/peerj.14316 (PMC9653067; doi:10.7717/peerj.14316)
Supplement: Supplemental Information 2 [file peerj-10-14316-s002.docx]

| Spearman’s correlation（p-value） | History of DM | Causes of renal failure | MLR | NLR | RDW | |
| --- | --- | --- | --- | --- | --- | --- |
| History of DM | 1 | -0.234(<0.001) | - | - | - |  |
| Causes of renal failure |  | 1 | - | - | - | |
| MLR | - | - | 1 | 0.625(<0.001) | 0.132(0.008) | |
| NLR | - | - | - | 1 | 0.206(<0.001) | |
| RDW | - | - | - | - | 1 | |

Abbreviations: DM, diabetes mellitus; MLR, monocyte/lymphocyte ratio; NLR, neutrophil/lymphocyte ratio; RDW, red blood cell distribution width; -, not applicable.
