# Supplementary figures and images for: Development of a prediction model to estimate the 5-year risk of cardiovascular events and all-cause mortality in haemodialysis patients: a retrospective study
Source: PeerJ. 2022 Nov 9;10:e14316. doi: 10.7717/peerj.14316 (PMC9653067; doi:10.7717/peerj.14316)

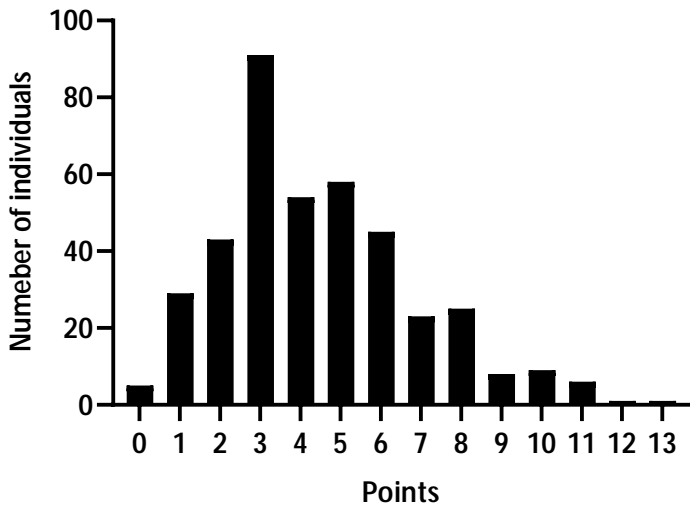

Supplement: Supplemental Information 3 [file peerj-10-14316-s003.pdf]

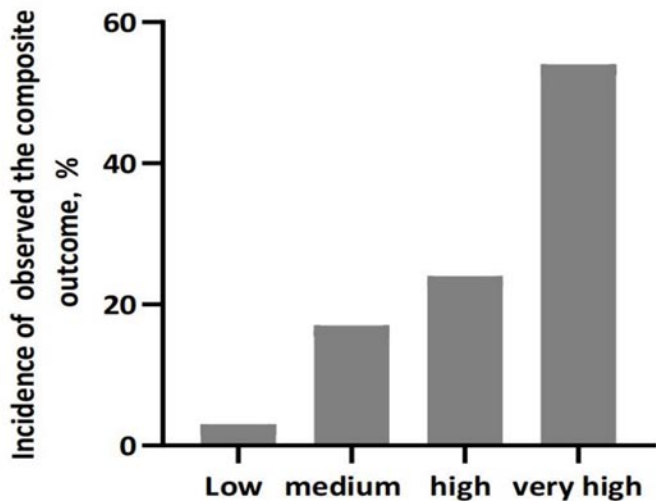

| Categories     | Low risk | Medium Risk | High Risk | Very high risk |
|----------------|----------|-------------|-----------|----------------|
| Score (points) | 0-2      | 3-4         | 5-6       | 7-13           |
| Incidence (%)  | 3.23     | 17.4        | 24.5      | 54.8           |

Supplement: Supplemental Information 4 [file peerj-10-14316-s004.pdf]

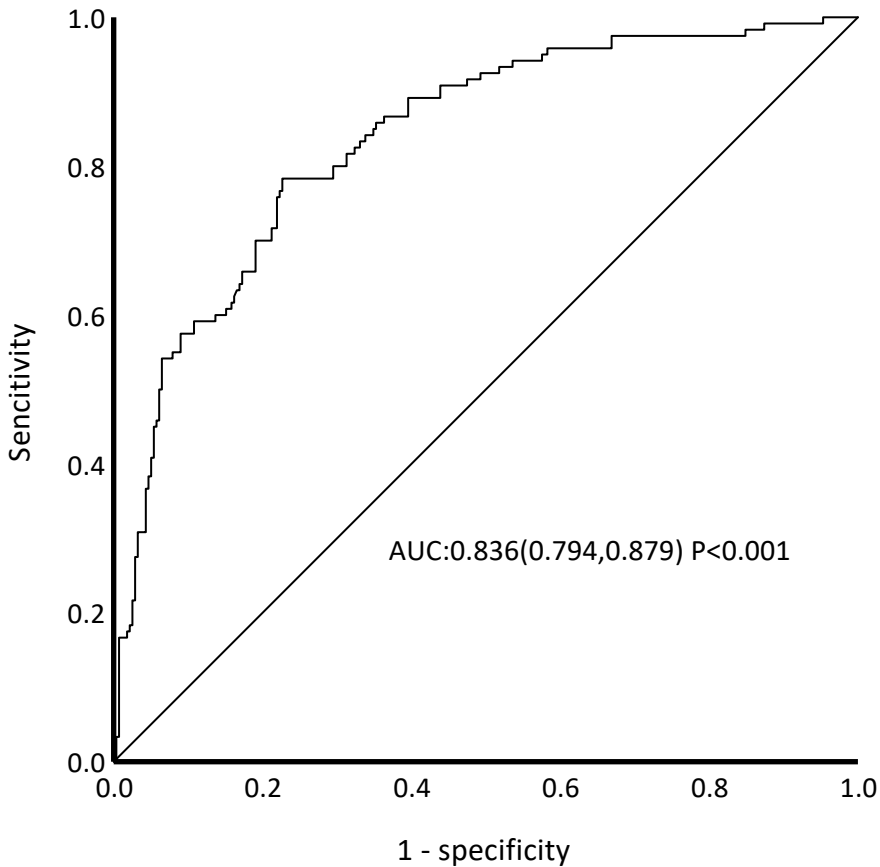

Supplement: Supplemental Information 5 [file peerj-10-14316-s005.pdf]

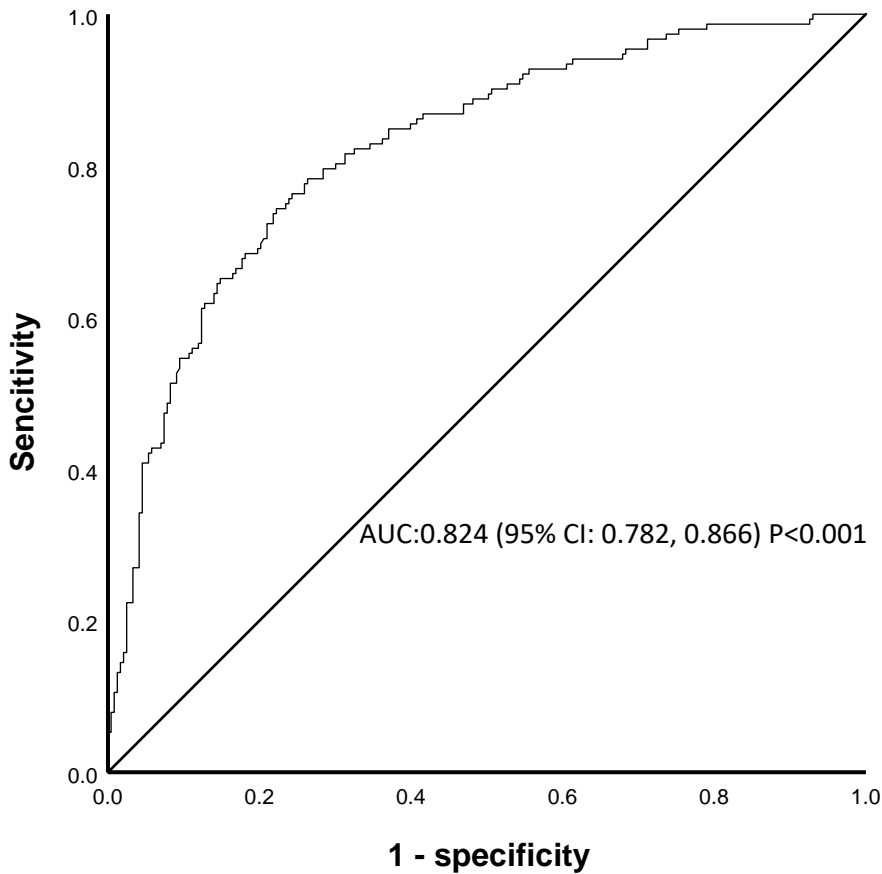

Supplement: Supplemental Information 6 [file peerj-10-14316-s006.pdf]

**A**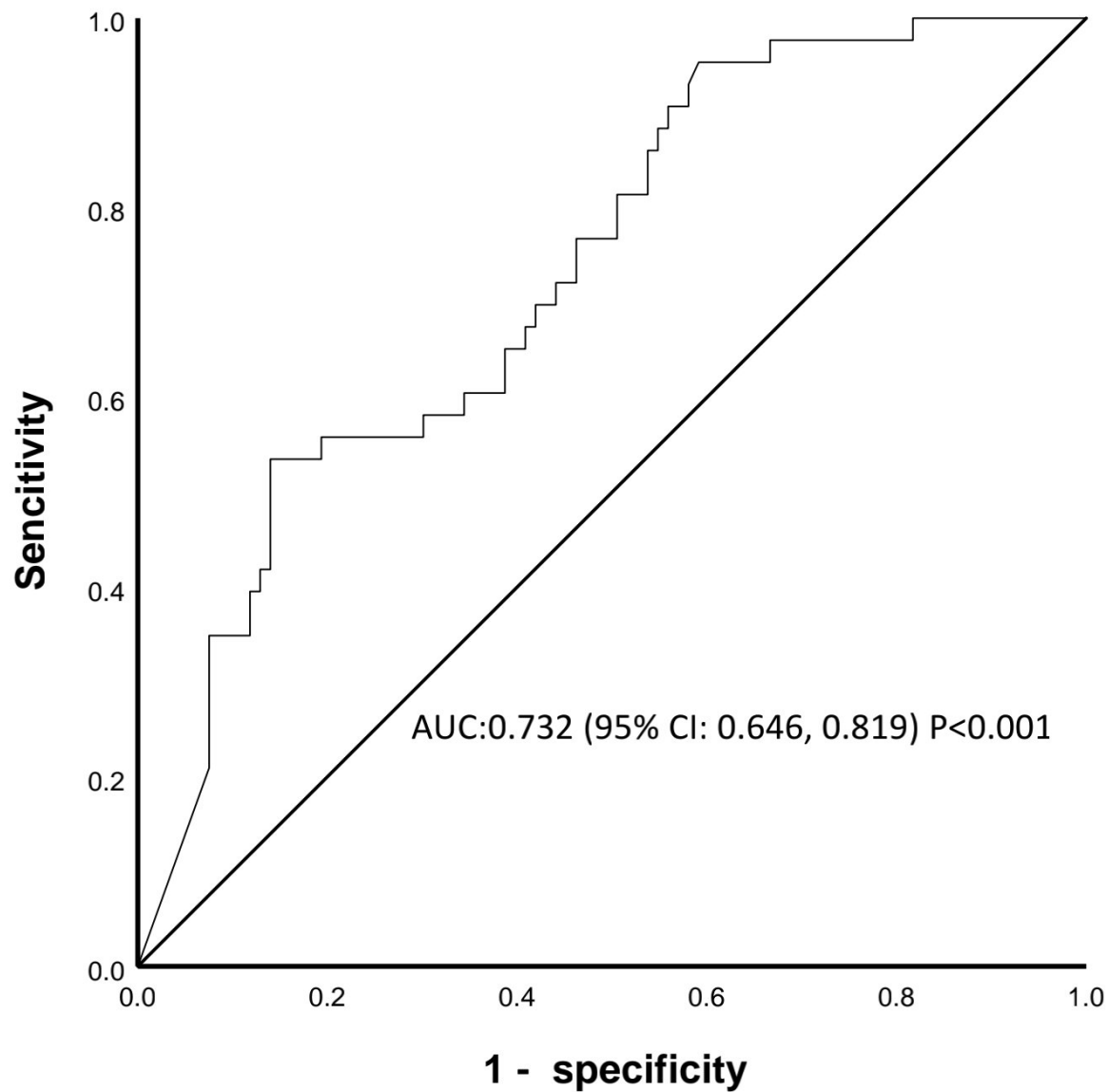

**B**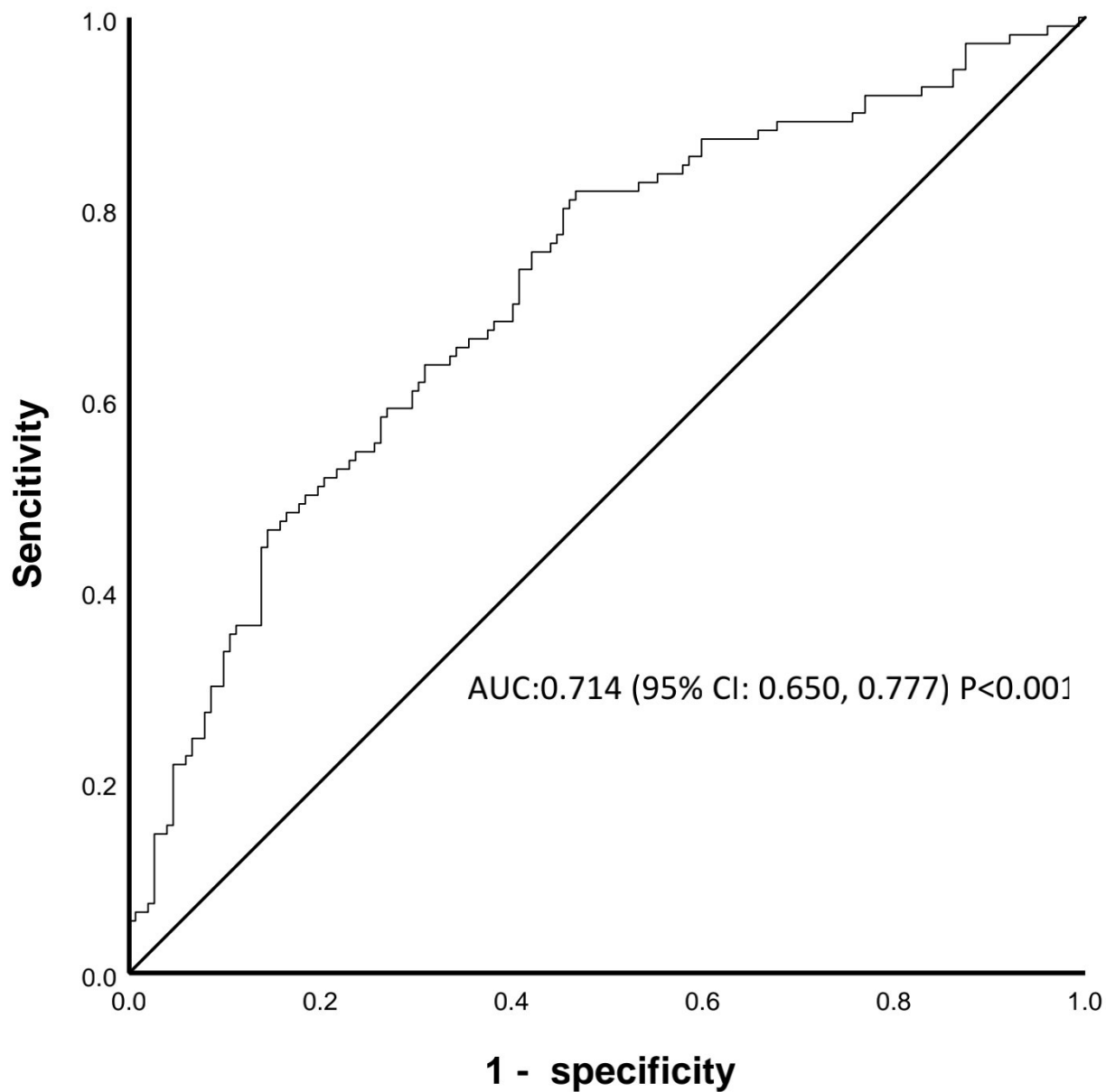

Supplement: Supplemental Information 7 [file peerj-10-14316-s007.pdf]
